# Bloodstream Infection and Associated Factors at University of Gondar Comprehensive Specialized Hospital, Northwest Ethiopia, 2023: A Prospective Cross‐Sectional Study

**DOI:** 10.1155/bmri/7745297

**Published:** 2025-12-01

**Authors:** Andinet Azaje Alemu, Aynishet Adane, Kassaye Demeke Altaye, Ayanaw Guadie Mamo, Hiber Asteraye Tsigie, Meseret Mulu, Asrat Elias Ergena, Daniel Belay Agonafir, Faisel Dula Sema, Abdisa Gemedi Jara

**Affiliations:** ^1^ Department of Internal Medicine, School of Medicine, College of Medicine and Health Sciences, University of Gondar, Gondar, Ethiopia, uog.edu.et; ^2^ Department of Emergency and Critical Care Medicine, School of Medicine, College of Medicine and Health Sciences, University of Gondar, Gondar, Ethiopia, uog.edu.et; ^3^ Department of Laboratory, School of Medicine, College of Medicine and Health Sciences, University of Gondar, Gondar, Ethiopia, uog.edu.et; ^4^ Department of Pharmaceutical Chemistry, School of Pharmacy, College of Medicine and Health Sciences, University of Gondar, Gondar, Ethiopia, uog.edu.et; ^5^ Department of Internal Medicine, School of Medicine, College of Medicine and Health Science, Wachemo University, Hossana, Ethiopia, wcu.edu.et; ^6^ Department of Clinical Pharmacy, School of Pharmacy, College of Medicine and Health Sciences, University of Gondar, Gondar, Ethiopia, uog.edu.et

**Keywords:** bacteria, bloodstream infection, contaminants, medical patients, UOGCSH

## Abstract

**Introduction:**

Bloodstream infections (BSIs) are the presence of circulating microorganisms in the bloodstream. Globally, the distribution and factors that influence BSIs are changing, which is an alarming sign to investigate. In addition, prospective data are limited in Ethiopia. For these reasons, it is necessary to assess the BSI and associated factors.

**Objectives:**

This study was aimed at assessing BSI and associated factors at the University of Gondar Comprehensive Specialized Hospital (UOGCSH), 2023.

**Methods:**

A prospective cross‐sectional study was conducted from August 2023 to December 2023 among 252 patients. The data was collected using consecutive sampling techniques, coded, and analyzed using SPSS Version 27. Multivariable binary logistic regression was used for variables with a *p* value of < 0.2 on bivariable binary logistic regression. Adjusted odds ratio (AOR) with 95% CI was used to report the strength of the association, and *p* value < 0.05 was used to declare a statistically significant association. The Hosmer and Lemeshow tests were used to confirm the goodness of fit of the model (*p* value, 0.734).

**Results:**

A total of 228 participants were included in this study, with a mean age of 41 (±18) years. Overall, bacterial growth was detected on 41 (18%) of blood cultures. Of these, 15 (6.6%, 95% CI: 3.5–9.6) were true BSI, while the remaining 26 (11.4%) were contaminants*. Klebsiella pneumoniae* was the most commonly detected bacterium. Blood volume, stroke, and neutrophil‐to‐lymphocyte count ratio (NLCR) are significantly associated with BSI, whereas poor venipuncture antiseptic techniques and being febrile are significantly associated with contaminants.

**Conclusion:**

Prevalence of true BSI is low, and collected blood volume, stroke, and high NLCR were associated with BSIs at UOGCSH. Training on blood sample collection, quality checks, and testing anaerobic bacteria and fungi is recommended.

## 1. Introduction

Blood is a sterile environment, but there could be transient and sporadic translocation of commensal microbes [1]. The presence of bacterial or fungal viable microorganisms in the bloodstream is called bloodstream infections (BSIs) [2]. BSIs are either primary without a defined focus of infection or secondary to a focal infection [3–5]. Bacteria account for the majority of BSI cases, while fungi, parasites, and viruses are less frequent [6, 7].

Globally, BSIs affect about 30 million people and account for 6 million deaths. It is the leading cause of morbidity and mortality throughout the world [8, 9]. In addition, BSIs are associated with prolonged hospitalization [10, 11] and a significant economic burden [3–5, 7]. BC is a key diagnostic tool for appropriate identification, diagnosis, early initiation, or modification of treatment of BSI, which is crucial in decreasing prolonged hospitalization and mortality [12, 13]. At least two BC sets are recommended to detect BSI [9, 14, 15]. Although up to a 30% positivity rate of BC is reported, only a small proportion of BCs (3.6%–10%) yield clinically significant pathogens [12, 16, 17]. Volume of blood and sample collection techniques are the most important determinants of BC results [14, 18, 19].

BSI ranges from 9.8% to 30% [6, 16, 20–22]. In Ethiopia, the pooled prevalence of BSIs was 25.78% [23]. Additional studies conducted at the University of Gondar Comprehensive Specialized Hospital (UOGCSH) reported that the prevalence of BSIs was 16.3% [24], 24.2% [25], 25% [26], 32% [27], and 44% [28]. *Klebsiella pneumonia* [24, 26], *Staphylococcus aureus* [27], and CoNS [25, 28] were the most commonly isolated bacteria, and the identified BSIs are usually treated with appropriate antimicrobial agents based on the sensitivity profile.

BSIs are a major public health concern, causing illnesses and deaths in low‐ and middle‐income countries, including Ethiopia [29]. Demographic changes, the emergence of multidrug‐resistant pathogens, advancements in medicine with increasing numbers of immunocompromised patients, and utilization of invasive devices are influencing the epidemiology of BSI [2, 6]. In addition, lesser or nonpathogenic CoNs are the major cause of nosocomial infections [30]. For this reason, assessing the prevalence, causative organisms, and associated factors of BSI is crucial. To the investigators′ knowledge, most studies conducted in Ethiopia, including this setup, have been retrospective, which limits the findings of these studies in nature. Furthermore, most available studies do not investigate factors associated with BSI and contaminants among medical patients. For this reason, this study is the first of its type to be conducted prospectively and assess the prevalence and factors associated with BSI among hospitalized medical patients.

## 2. Method

### 2.1. Study Design and Setting

A cross‐sectional study was conducted from August 2023 to December 2023 at UOGCSH Northwest, Ethiopia. UOGCSH resides in Gondar City of Amhara Region, which is located 738 km from Addis Ababa (capital city of Ethiopia) and 172.8 km from Bahir Dar (capital city of Amhara Region). UOGCSH was established in 1954 as a public health college and currently serves as a referral center for more than 8 million people with around 960 inpatient beds. The internal medicine department is one of the major departments of UOGCSH and provides emergency, intensive care unit, inpatient, outpatient, and dialysis services with 200 beds.

### 2.2. Population

Patients admitted to UOGCSH medical wards (emergency, inpatient, and intensive care unit) during the prospective data collection period were considered the source populations. Participants admitted to these wards and their blood samples collected for BC were the study population.

### 2.3. Eligibility Criteria

All patients admitted to the medical wards of UOGCSH during the study period, willing to participate, aged ≥ 18 years, and for whom blood samples were collected for BC were included in this study. However, patients who refused to participate, patients with incomplete BC prescriptions, and BC samples collected from other wards were excluded from this study.

### 2.4. Sample Size Determination and Sampling Method

The sample size was determined using a single population proportion formula, *N* = (z*α*/2)^2^ *P*(1 − *p*)/(*d*)^2^, where *N* is the sample size, *P* is the proportion, and *d* is the margin of error. Then, a prevalence of BSI of 18.2% from a previous study conducted at UOGCSH [31], a level of confidence of 95%, and a 5% margin of error were used to calculate the sample size. After considering the 10% nonresponse rate, the final sample size was 252. The data was collected using a consecutive sampling technique.

### 2.5. Variable of the Study

BSIs were the dependent variables, whereas sociodemographic characteristics (age, sex, residency, and occupation), clinical characteristics (primary diagnosis, possible source of infection, comorbidities, vital signs, and antibiotic exposure status), laboratory values (complete blood cell count, neutrophil‐to‐lymphocyte count ratio [NLCR], creatinine, urea, liver enzymes, and bilirubin), and sample characteristics (skin preparation, volume of blood sample, number of BC bottles, anatomic sites of collection, patients′ site of admission, antiseptic technique, and timing of sample collection, febrile episodes, and antibiotic exposure) were treated as the independent variables.

### 2.6. Data Collection Instrument, Procedure, and Quality Control

The data was collected prospectively using an interviewer‐administered questionnaire and chart review. The data collection tool had five major parts developed by reviewing different available literature: sociodemographic and clinical characteristics of the patient [32–34], laboratory values [16], sample characteristics [15, 35], and BC result.

The data was collected by four trained nurses after receiving training on the study objective, methodology, data collection method, confidentiality of information, participants′ rights, and ethical aspects. The questionnaire was translated into Amharic (a local language) for a better understanding of the study participants and back‐translated to English to minimize potential translation errors. The collected data were reviewed and checked for completeness, accuracy, and consistency by the supervisor and principal investigators.

Blood volume was measured using a calibrated syringe, and appropriate tubes were used to transfer the collected blood. Laboratory values (complete blood cell count, NLCR, creatinine, urea, liver enzymes, and bilirubin) were extracted from the patient′s laboratory results. Sample characteristics were also collected from patient charts and through a checklist.

### 2.7. Operational Definitions

Samples with one positive BC result for microorganisms were considered true positives, except CoNs, *Corynebacterium* species, *Bacillus* species (except *Bacillus anthracis*), and *Micrococcus* species or enterococci and viridian streptococci.

CoNs, *Corynebacterium* species, *Bacillus* species (except *B. anthracis*), and *Micrococcus* species or enterococci and viridian streptococci were considered true positives when two or more positive BC results of the same species from samples taken at different body sites or different time intervals with appropriate clinical correlation were found [14, 33, 36].

Patients with true positive BC results were considered, as they have BSIs, whereas positive results that do not fulfill the true positive criteria were contaminants or false positives.

### 2.8. Data Processing and Analysis

The data that passed the quality check were coded, entered into EpiData Version 4.6, and analyzed using SPSS Version 27. Categorical variables were summarized as frequency (percentage) of the total. The Kolmogorov–Smirnov test was used to assess the distribution of continuous variables, and normally distributed variables were expressed as mean (±SD). Multicollinearity of independent variables was tested using the chi‐square test. For variables without multicollinearity, and significantly associated (*p* value < 0.2) on bivariable binary logistic regression, multivariable binary logistic regression was run to identify factors associated with BSI. Adjusted odds ratio (AOR) with 95% CI was used to report the strength of the association, and *p* value < 0.05 was used to declare a statistically significant association. The goodness of fit of the model was confirmed by the Hosmer and Lemeshow test (*p* value, 0.734).

## 3. Result

### 3.1. Sociodemographic Characteristics of Participants

A total of 228 study participants were included in this study, giving a response rate of 90.5%. Above half of the participants were males (55.7%), with a male‐to‐female ratio of 1.26. The mean age of participants was 41 (±18) years, and the majority were rural dwellers (*n* = 132; 57.3%) (Table 1).

**Table 1:** Sociodemographic characteristics of hospitalized medical patients at UOGCSH, Northwest Ethiopia, 2023.

**Variable**	**Categories**	**Number (%)**
Age	16–40	128 (56.1)
	41–59	56 (24.6)
	≥ 60	44 (19.3)

Mean (± SD) age	41 (±18)	

Sex	Female	101 (44.3)
	Male	127 (55.7)

Residency	Rural	132 (57.3)
	Urban	96 (42.7)

Marital status	Single	54 (23.7)
	Married	143 (62.7)
	Divorced	18 (7.9)
	Widowed	13 (5.7)

Occupation	Jobless	72 (31.6)
	Farmer	101 (44.3)
	Civil servant	24 (10.5)
	Private business	18 (7.9)
	Day laborer	13 (5.7)

Religion	Orthodox Christian	207 (90.8)
	Muslim	11 (4.8)
	Protestant Christian	10 (4.4)

### 3.2. Clinical and Sample Characteristics

Pneumonia was the most common source of BSIs (*n* = 12). More than two‐thirds (161, 70.6%) of the participants experienced febrile episodes within 24 h, and 199 (87.3%) received antibiotics before the blood sample was collected. Then, 70% alcohol was commonly used as an antiseptic (174, 76.3%), and 191 (83.8%) participants′ blood samples were collected after 24 h of hospitalization. The mean volume of collected blood samples was 15.43 mL (±4.38) (Table 2). Ceftriaxone was the most commonly administered antibiotic, followed by vancomycin and metronidazole. For the majority (96.5%) of participants, a blood sample was collected using two aerobic BC bottles.

**Table 2:** Clinical characteristics and collected sample characteristics of hospitalized medical patients at UOGCSH, Northwest Ethiopia, 2023.

**Variables**		**True positive**	**Negative/false positive**	**Total**
Clinically suspected source of BSI	Pneumonia	12	101	113
	Gastrointestinal tract	1	21	22
	Unidentified source	2	91	93

Comorbidities	No comorbidities	4	65	69
	Hypertension	4	40	44
	Diabetes mellitus	3	20	23
	Stroke	6	29	35
	Retroviral infection	1	19	20
	Hematologic diseases	1	40	41
	Others^a^	0	16	16

Antibiotics given before BC	No	0	29	29 (12.7)
	Yes	15	184	199 (87.3)

Febrile episode within 24 h before blood collection	No	7	60	67 (29.4)
	Yes, maximum < 38.5	2	77	79 (34.6)
	Yes, maximum ≥ 38.5	6	76	82 (36)

Timing of BC after hospitalization	≤ 24 h	1	36	37 (16.2)
	> 24 h	14	177	191 (83.8)

Blood draw timing	Day	12	186	198 (86.8)
	Night	3	27	30 (13.2)

Site of venipuncture	Same	5	80	85 (37.3)
	Different	9	134	143 (62.7)

Antiseptics	Not used	0	18	18 (7.9)
	70% alcohol	13	161	174 (76.3)
	Povidone iodine	2	27	29 (12.7)
	Chlorhexidine	0	7	7 (3.1)

Amount of blood	Mean (mL)	17.73	15.26	15.43

White blood cell count	Mean	11.92	11.97	11.97
	≤ 12,000	9	154	163 (71.5)
	> 12,000	6	59	65 (28.5)

NLCR	Mean	12.2	7.7	8

Creatinine	Mean	1.08	1.27	1.25
	≤ 1	7	125	132
	> 1	6	71	77

Abbreviations: BC, blood culture; BSI, blood stream infection; NLCR, neutrophil‐to‐lymphocyte count ratio.

^a^Other (chronic kidney disease, chronic liver disease, malignancy, and respiratory disease).

### 3.3. Blood Culture

**Figure 1:**
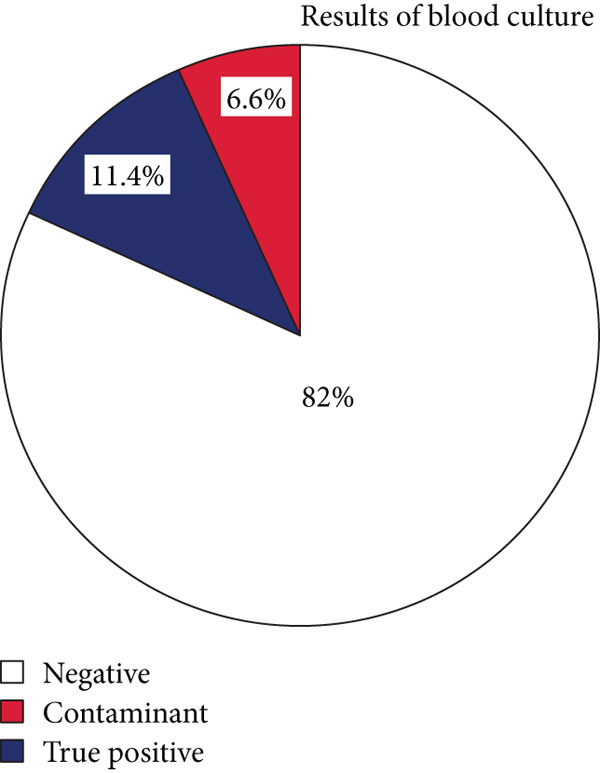
Blood culture distribution among different medical wards at UOGCSH, Northwest Ethiopia, 2023.

Bacterial growth was detected on 41 BC samples (18%, 95% CI: 13.6–23.7). However, only 15 (6.6%, 95% CI: 3.5–9.6) of them were true positives (BSI) (Figure 1), and 80% of them were gram‐negative bacteria. *Klebsiella pneumoniae* (*n* = 4; 26.7%) is the most commonly identified BSI bacterium, followed by *Escherichia coli* (*n* = 3; 20%), nonlactose fermenter gram‐negative rods (NLFGNR) (*n* = 3; 20%), and *Pseudomonas aeruginosa*, *Streptococcus pneumoniae*, *Citrobacter*, CoNS, and other undifferentiated *Streptococcus* species each account for 6.7% of BSI.

**Figure 2:**
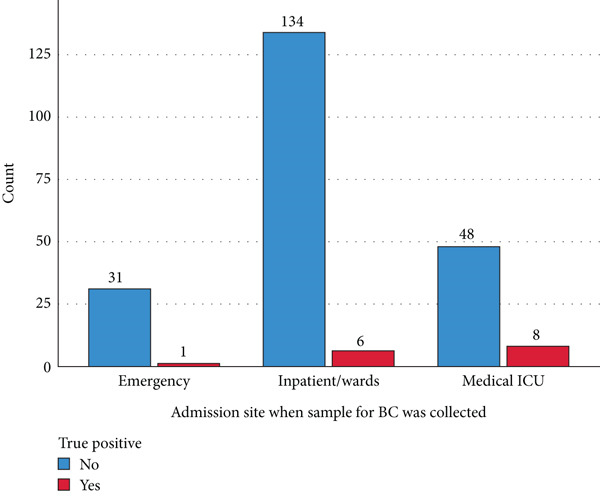
Blood culture distribution among different medical wards at UOGCSH, Northwest, Ethiopia, 2023.

The majority of study participants were from medical wards (*n* = 140; 61.4%), and over half (*n* = 8, 53.3%) of BSIs were detected in the intensive care unit (Figure 2).

### 3.4. Factors Associated With the Blood Culture Positivity Rate

The multivariable binary logistic regression was used to identify factors associated with BSI, and the volume of collected blood, NLCR, and stroke were associated with BSIs. Patients drawn with ≥ 16 mL of blood, stroke patients, and patients with NLCR greater than 12 have 8.56 times (AOR = 8.56, 95% CI: 1.455–50.301, *p* = 0.018), six times (AOR = 5.92, 95% CI: 1.329–26.397, *p* = 0.020), and 4.5 times (AOR = 4.468, 95% CI: 1.047–19.057, *p* = 0.0437) higher odds of getting true positive BC, respectively (Table 3).

**Table 3:** Factors associated with BSI among hospitalized medical patients at UOGCSH, Northwest Ethiopia, 2023.

**Variables**	**Categories**	**BC result,** **n** ** (%)**		**COR (95% CI)**	**AOR (95% CI)**	**p** ** value**
		**True positive**	**Negative/contaminant**			
Age	16–40	4 (3.1)	124 (96.9)			
	41–59	6 (10.7)	50 (89.3)	3.720 (1.007–13.747)	3.795 (0.779–18.491)	0.099
	≥ 60	5 (11.4)	39 (88.9)	3.974 (1.017–15.533)	1.067 (0.156–7.319)	0.947

Sex	Female	4 (4)	97 (96)			
	Male	11 (8.7)	116 (91.3)	2.300 (0.710–7.452)	2.482 (0.589–10.453)	0.215

Admission	Emergency	1 (3.1)	31 (96.9)			
	Ward	6 (4.3)	134 (95.7)	1.388 (0.161–11.949)	0.948 (0.077–11.712)	0.966
	ICU	8 (14.3)	48 (85.7)	5.167 (0.616–43.361)	2.460 (0.199–30.470)	0.483

Pneumonia	No	3 (2.6)	112 (97.4)			
	Yes	12 (10.6)	101 (89.4)	4.436 (1.217–16.169)	3.809 (0.863–16.816)	0.078

DM	No	12 (5.6)	201 (94.4)			
	Yes	3 (20)	12 (80)	4.187 (1.040–16.859)	4.214 (0.562–31.582)	0.162

Stroke	No	9 (4.7)	184 (95.3)			
	Yes	6 (17.1)	29 (82.9)	4.230 (1.401–12.767)	5.132 (1.206–21.842)*	0.027*

Febrile episode within 24 h	No	7 (10.4)	60 (89.6)			
	Yes, ≤ 38.5	2 (2.5)	77 (97.5)	0.223 (0.045–1.111)	0.229 (0.035–1.507)	0.125
	Yes, > 38.5	6 (7.3)	76 (92.7)	0.677 (0.216–2.120)	1.176 (0.272–5.076)	0.828

Amount of blood collected	< 16 mL	2 (1.9)	101 (98.1)			
	≥ 16 mL	13 (10.4)	112 (89.6)	5.862 (1.291–26.608)	8.555 (1.455–50.301)*	0.018*

NLCR	≤ 12	9 (4.9)	174 (95.1)			
	> 12	6 (14.3)	36 (85.7)	3.222 (1.080–9.618)	4.468 (1.047–19.057)*	0.043*

*Note:* Bold data shows statistically significant values.

Abbreviations: AOR, adjusted odds ratio; BC, blood culture; CI, confidence interval; COR, crude odds ratio; DM, diabetes mellitus; ICU, intensive care unit; NLCR, neutrophil‐to‐lymphocyte count ratio.

*Statistically significant.

To determine the factors associated with CoNS, a binary logistic regression was fitted, and the odds of obtaining CoNS were lower by 81% when samples were collected using an appropriate antiseptic technique (AOR = 0.178, 95% CI: 0.046–0.696, *p* = 0.013). However, the odds of getting CoNS were 15 times higher among febrile patients (≥ 38.5°C) (AOR = 15.414, 95% CI: 1.86–22.49, *p* = 0.011).

## 4. Discussion

A prospective cross‐sectional study was aimed at assessing the prevalence of BSIs, and associated factors among hospitalized medical patients was conducted at UOGCSH. The overall BC positive yield was 18% (95% CI: 13.2–23.6), which is in line with previously conducted studies at UOGCSH (19.7%) [28], Nigeria (18.2%) [37], Italy (17.2%) [12], Nepal (23.1%) [38], and Brazil [10]. However, this result is higher than a study conducted at Arba Minch (9.8%) [20] and lower than studies conducted at Mekelle (28%) [39], Addis Ababa (30.5%) [22], and Gambia (34%) [40]. This difference might be observed due to differences in sample size, study design, source population, geographical location, and detection rate of contaminants. In addition, differences in blood sample collection and BC preparation techniques used might also contribute to the observed difference.

A total of 15 (6.6%, 95% CI: 3.7–10.6) BSIs were identified, and this was in line with a study conducted at UOGCSH (10.5%) [31] and the Netherlands (6.4%) [16]. However, it was lower than a study conducted in Italy (11.5%) [12]. This difference may arise from differences in source populations, specimen quality, different infection control policies, and bacteriological techniques used to detect BSI. Overall, the lack of testing for anaerobic bacteria, fungi, and blood sample drawing for culture after antibiotics administration might be attributed to the observed lower BSI rate in this study.

In this study, only one contaminant (3.7%) was truly positive, which is lower than the study conducted in India (24.1%) [36]. This difference may be observed due to variation in blood sample collection technique and limited practice of repeating BC tests for common contaminants as per guidelines. Two positive BC results of contaminants are a standard recommendation to identify true positive pathogens [14]. A statistically significant association was observed between CoNS and febrile patients (≥ 38.5°C) within 24 h before the blood sample was collected in this study. This might show that CoNS can be more true positive than reported if BC was repeated and appropriate clinical correlation and time to positivity were considered [36]. Available evidence also showed that even though CoNS are among the commonly identified contaminants [41] and less or nonpathogenic, currently, they are the major cause of nosocomial infections [30]. Since CoNS have a significant impact on human health and well‐being, all recommended techniques should be implemented as per guidelines to identify clinically significant CoNS.

On the other hand, this study found that 11.4% (95% CI: 7.6–16.3) of positive results were contaminants. This is in line with studies conducted previously in Gondar, Ethiopia (7.7%) [31], and Addis Ababa (9.1%) [22]. However, this finding is higher than studies conducted in Germany (2.8%) [6], the Netherlands (6.4%) [16], and the recommended rate of contamination (3%) by the American Society for Microbiology and the Clinical Laboratory Standards Institute [42]. These differences might be attributed to poor venipuncture techniques in this study setting. In addition, this study also revealed that poor practice of antiseptic techniques leads to the detection of contaminants in BCs. Using standard operating procedures for blood sample collection and BC preparation can reduce the prevalence of contaminants [43].

BSIs are not affected by age, and similar findings were also reported in a study conducted in Mekelle [39]. However, opposite findings were also reported in studies conducted in Gondar, Ethiopia [26, 31], and Nigeria [44]. This difference might be observed due to the difference in the source population, as this study does not include the pediatric population.

The study also found that patients who had ≥ 16 mL of blood collected were more likely to have a BSI compared to those with less than 16 mL of blood (AOR = 8.56, 95% CI: 1.455–50.301, *p* = 0.018). A similar finding was also reported in Sweden [18]. Other available evidence also supported that the collected blood volume is known as the single most important determinant of blood culture yield [18, 45–48]. In addition, this study revealed that stroke patients were more likely to have a BSI (AOR = 5.92, 95% CI: 1.329–26.397, *p* = 0.02). Pieces of evidence also supported that the risk of BSI is higher among stroke patients [49]. Pneumonia was a clinically presumed source of BSIs for stroke patients in this study, and other conducted systematic reviews and meta‐analyses also reported that pneumonia is among the most common sources of BSIs among stroke patients [50].

Patients with NLCR > 12 have been found to have a greater chance of getting BSIs than patients with NLCR < 12, which is similar to the study conducted in Sweden [51]. Another comparative study also showed that NLCR is higher among patients with BSI, and an increment in NLCR is associated with a high chance of BSIs [52]. Available shreds of evidence also reported that in patients with BSIs, WBC and neutrophil counts increase, while depletion of lymphocytes leads to an elevated NLCR ratio [53, 54].

## 5. Strengths and Limitations of the Study

Since the study is prospective cross‐sectional, the biases are minimized, as relatively similar BC methods were employed by the same laboratory technicians. This reduces differences and gives consistent procedures. However, this study is not conducted without any limitations. The relatively smaller sample size could negatively affect the accuracy and precision of detecting associations for variables with smaller effect sizes, and being a cross‐sectional study limits the determination of the cause‐and‐effect relationship. A large proportion had prior antibiotic exposure, and culture was not done for anaerobic bacteria, which can significantly reduce BSI detection. In addition to the aforementioned limitation, the single‐center nature limits its generalizability.

## 6. Conclusion and Recommendation

The overall culture positivity rate is high. However, the yield of clinically significant bacteria among hospitalized medical patients at UOGCSH is low due to the high rate of contaminants. The majority of identified BSI microorganisms were gram‐negative bacteria. Collected blood volume, stroke, and high NLCR were significantly associated with BSIs. Inappropriate antiseptic techniques during venipuncture are associated with a higher rate of contamination. Despite being contaminants, they have a significant association with febrile episodes and should be carefully evaluated as possible BSIs.

Collecting an adequate amount of blood and using appropriate antiseptics is necessary to increase the yield of true pathogens and decrease the contamination rate, respectively. Continuous training on blood sample collection for BC and quality checks of collected samples is recommended. To determine the real‐time prevalence of BSI, future research should also investigate BSI caused by anaerobic bacteria and fungi.

**Nomenclature**
AOR: adjusted odds ratio
BC: blood culture
BSIs: bloodstream infections
CI: confidence interval
CoNS: coagulase‐negative staphylococci
NLCR: neutrophil‐to‐lymphocyte count ratio
UOGCSH: University of Gondar Comprehensive Specialized Hospital
WBC: white blood cell count

## Ethics Statement

The proposal was submitted to the Department of Internal Medicine, and ethical clearance was obtained from the University of Gondar, Department of Internal Medicine, Research and Ethical Review Committee. In addition, a letter of cooperation was submitted to UOGCSH. Moreover, the purpose of the study was explained, and written informed consent was obtained from the study participants and their legal guardians. Confidentiality was maintained at all stages of the study by omitting participants′ names and other identifiers. This study was conducted in accordance with the Helsinki Declaration.

## Disclosure

All authors gave final approval of the version to be published, have agreed on the journal to which the article has been submitted, and agree to be accountable for all aspects of the work.

## Conflicts of Interest

The authors declare no conflicts of interest.

## Author Contributions

All authors (A.A.A., A.A., K.D.A., A.G.M., H.A.T., M.M., A.E.E., D.B.A., F.D.S., and A.G.J.) made a significant contribution to the work reported, whether that is in the conception, study design, execution, acquisition of data, analysis, and interpretation, or all these areas, and took part in drafting, revising, or critically reviewing the article.

## Funding

This study was funded by the College of Medicine and Health Sciences, University of Gondar (10.13039/501100007866).

## Data Availability

The datasets used and/or analyzed during the current study are available from the corresponding author upon reasonable request.
